# Characterization of cross protection of Swine-Origin Influenza Virus (S-OIV) H1N1 and reassortant H5N1 influenza vaccine in BALB/c mice given a single-dose vaccination

**DOI:** 10.1186/1423-0127-20-19

**Published:** 2013-03-21

**Authors:** Hui-Tsu Lin, Chuan-Chang Chuang, Hsieh-Ling Wu, Der-Ming Chu, Yeau-Ching Wang

**Affiliations:** 1 Institute of Preventive Medicine, National Defense Medical Center, PO Box 90048–700, San-Hsia, Taiwan; 2 Tri-Service General Hospital, National Defense Medical Center, Taipei, 100, Taiwan

**Keywords:** S-OIV, H1N1, H5N1, Adjuvant, Alum, MPLA

## Abstract

**Background:**

Influenza virus has antigen drift and antigen shift effect, vaccination with some influenza vaccine might not induce sufficient immunity for host to the threat of other influenza virus strains. S-OIV H1N1 and H5N1 influenza vaccines in single-dose immunization were evaluated in mice for cross protection to the challenge of A/California/7/2009 H1N1 or NIBRG-14 H5N1 virus.

**Results:**

Both H1N1 and H5N1 induced significant homologous IgG, HAI, and microneutralization antibody responses in the mice, while only vaccines plus adjuvant produced significant heterogeneous IgG and HAI antibody responses. Both alum and MPLA adjuvants significantly reduced the S-OIV H1N1 vaccine dose required to elicit protective HAI antibody titers from 0.05 μg to 0.001 μg. Vaccines alone did not protect mice from challenge with heterogeneous influenza virus, while H5N1 vaccine plus alum and MPLA adjuvants did. Mouse body weight loss was also less significant in the presence of adjuvant than in the vaccine without adjuvant. Furthermore, both H1N1 and H5N1 lung viral titers of immunized mice were significantly reduced post challenge with homologous viruses.

**Conclusion:**

Only in the presence of MPLA adjuvant could the H5N1 vaccine significantly reduce mouse lung viral titers post H1N1 virus challenge, and not vice versa. MPLA adjuvant induced cross protection with a single dose vaccination to the challenge of heterogeneous influenza virus in mice. Lung viral titer seemed to be a better indicator compared to IgG, neutralization antibody, and HAI titer to predict survival of mice infected with influenza virus.

## Background

The swine-original influenza virus A (S-OIV) H1N1 was determined to be a novel strain of A/Influenza H1N1 serotype which had been derived by reassortment of swine, avian and human influenza viruses. The WHO declared that the infections caused by the new strain had reached pandemic proportions on June, 2009; and has reported approx. 14700 deaths in more than 209 countries resulting from pandemic influenza H1N1 [1].

Immunization provides the best preventive strategy against influenza virus illness. The current trivalent vaccine is unlikely to provide significant protection against the novel pandemic H1N1 strain, especially for children and young adults because of absence or low immunity to the novel 2009 H1N1 strains [2,3]. It has been reported that previous vaccination of children with trivalent vaccine of the last four seasons did not elicit a cross-reactive antibody response to the pandemic H1N1 strain [4]. Thus, a monovalent vaccine based on the novel H1N1 strain will be required to induce protective immunity.

Current influenza virus vaccines aim to induce strong antibody (Ab) responses to the ectodomains of hemagglutinin (HA) and neuraminidase (NA) molecules, since these antibodies (Abs) can provide potent protection against infection and/or disease. The main deficiency of this protection is that it targets highly variable viral determinants. Failure to anticipate the emergence of an epidemic strain with significant antigenic changes compared to the vaccine strain will greatly reduce vaccine-induced protection. Several studies have suggested that proper adjuvant might improve the immunity of influenza vaccine and reduce the dose of vaccine [5-10]. Aluminum hydroxide (alum) is currently the only human vaccine adjuvant approved for use in the United States, and although it is effective at boosting antibody responses, these responses require repeated administration and tend to generate antiparasitic T helper 2 (T_H_2), rather than antiviral and antibacterial T_H_1, T cell immunity [11]. As a consequence, there is much effort devoted to develop prospective adjuvant that can establish protective immunity with fewer vaccinations and less injected material, through durable antibody and T_H_1-dependent cytotoxic T cell activity. Other potential immune adjuvant might be considered and developed. As demonstrated previously, Monophosphoryl lipid A (MPLA) is a low-toxicity derivative of LPS with useful immunostimulatory properties, which is nearing regulatory approval for use as a human vaccine adjuvant. Most recently, it has been demonstrated that the use of Al(OH)_3_ with MPLA as an emulsion induced a further increase in HAI titers of A/reassortant/NIBRG-14/Viet Nam 1194/2004 × Puerto Rico/8/1934 H5N1 (NIBRG-14) inactivated whole virus and split virion influenza vaccines [12]. These finding may have important implications for the development of future vaccine adjuvant.

Although there are studies evaluating immune response and protection of influenza H1N1 vaccine to 2009 S-OIV in ferrets and mice most recently [13-16]. Some studies also approached immune response and protection of vaccinated animals against other influenza virus in the absence or the presence of adjuvant. In these studies, some useful information has been revealed. For example, pandemic H1N1 vaccination is effective in mice [17]; the adjuvant MPLA can reduce the effective immunization dose of H5N1 and H3N2 influenza vaccines in mice [18]; and some other adjuvant can reduce the effective immunization dose of pandemic vaccines in mice [19,20]. However, most of these studies focus on the protection of BALB/c mice immunized in a two-dose regimen of vaccination. Since it is impossible to vaccinate people twice at the emergent time during influenza virus is pandemic worldwide, effective vaccine component and dosage of influenza vaccine for single immunization is more practical and critical for the prevention of influenza virus epidemic. In this study, single-dose immunization of 2009 H1N1 vaccine based on NIBRG-121 vaccine strain and avian H5N1 vaccine based on NIBRG-14 vaccine strain were evaluated for its effectiveness to elicit protective immunity of mice to the lethal challenge of A/California/07/2009 H1N1 virus. Meanwhile, the minimum effective dose of single immunization in the absence or the presence of adjuvant (either alum or MPLA); as well as the cross protection of heterogeneous vaccine, the changes of adjuvant-induced immune responses, and the reduction of vaccine dose used in the presence of adjuvant were also elucidated.

## Methods

### Vaccine strains and reagents

Influenza A virus NIBRG-121 (NIBSC, A/California/7/2009 H1N1 virus), virus (A/California/07/2009 H1N1; CDC# 2009712112), hemagglutinin (HA) antigen (A/California/7/2009, NIBSC 09/146), and antiserum (A/California/7/2009, NIBSC 09/152) were obtained from the Centers for Disease Control and Prevention (CDC, Taiwan), or the National Institute for Biological Standards and Control (NIBSC, UK). NIBRG-121 was used as vaccine strain, and virus A/California/07/2009 H1N1 was used as challenge virus.

### Vaccine production

NIBRG-121 virus strain was amplified in embryonic eggs of 10-day special pathogen free (SPF) for 48 h, it was then harvested and inactivated with 0.04% formalin at 4°C for overnight. Harvested virus was then filtered through 0.45 μm filter and concentrated with Lab scale TM TFF System, treatment with benzonase (cat. no. 1.01656.0001, Merck KGaA, Germany) (9 ~ 90 u/ml at 4°C for overnight), and purified with 10-50% sucrose gradient by Alfa Wassermann PKII Pilot-Scale Ultracentrifuge System. Purified virus was dialyzed with PBS, filtered with 0.45 μm filter and stocked in 1.5-ml stock tube at −80°C until use. Hemagglutinin (HA) antigen concentration was determined by single radial immunodiffusion assay (SRD) [21] for the quantification of vaccine S-OIV H1N1.

### Quality control assays of vaccine

To monitor the quality of produced vaccine, several items of assays were evaluated. Vaccine was quantified with SRD method; ovalbumin was evaluated with Chicken Egg Ovalbumin Elisa Kit (Cat. No. 6050) from Alpha Diagnostic International (ADI); endotoxin was measured with Toxin Sensor TM Chromogenic LAL Endotoxin Assay Kit (Cat. No. L00350) obtained from the GenScript Corporation. Above assays were followed the protocols of commercial kits. Formalin content of vaccine was evaluated according to the guideline of version 6^th^ of Pharmacopoeia Chinensis. For abnormal toxicity assay, male BALB/c mice (body weight: 17–22 gm) were inoculated intraperitoneal with 0.5 ml of produced vaccine (at least 5 mice per dose of vaccine) and then observed their changes of body weight. It fit the demand criteria only if there is no death, accident symptoms of mice, and mice should recovery of their body weight to the level of pre-inoculation 7 days after inoculation. The antigenicity of vaccine was evaluated with hemagglutination (HA) assay as described with HAI assay without antibody.

### Determination of vaccine HA concentration

The HA concentration of S-OIV candidate vaccine was measured with single-radial immunodiffusion (SRD) according the protocol described previously [22,23].

### Ethics statement

All animal experiments were reviewed by the Institutional Animal Care and Use Committee and approved by the regulatory authorities of Taiwan. All animal experiments were conducted in accordance with Taiwan laws on animal experimentation and guidelines set out by the Association for Assessment and Accreditation of Laboratory Animal Care International (AAALAC) and the Office of Laboratory Animal Welfare (OLAW). The IACUC certificate No. of this study were AN-99-05、 AN-100-07、 and AN-101-06. Animal were housed according to OLAW and AAALAC guidelines, in housing facilities accredited by the Center of Disease Center (CDC) of Taiwan.

### Immunization of mice

Female BALB/c mice (8-week-old) were immunized intraperitoneal with different dosage of produced vaccine (PBS control, 0.001 μg, 0.01 μg, and 0.05 μg) in the absence or presence of adjuvant alum (final 5% of Aluminum hydroxide gel, cat no.A8222, Sigma) or MPLA (Monophosphoryl Lipid A) (Sigma Adjuvant System, cat no.S6322, Sigma) using a single-vaccination regimen.

### IgG subclass determination

ELISA plates were coated with 10 ng HA per well of purified vaccine at 4°C for overnight. After blocking nonspecific binding (5% skim milk in PBST, 1 h at 37°C) and subsequent washing, PBST diluted mice sera (1:400) of pre-immune or immunized were added to wells and plated were incubated for 1 h at 37°C, washed again, prior to further incubation for 1 h with IgG subclass-specific peroxidase-conjugated goat or rabbit anti-mouse IgG antibodies (IgG: goat anti-mouse IgG (H + L) HRP, cat. no. 115-035-146, Jackson ImmunoResearch, USA; IgG1: rabbit anti-mouse IgG1 HRP, cat. no. 61–0120, Invitrogen, USA; IgG2b: rabbit anti-mouse IgG2a HRP, cat. no.61-0220, Invitrogen, USA). Bound IgG subclass antibodies were detected colorimetrically using TMB substrate by OD_450_ nm endpoint reading. The cutoff value was set at ODn plus three standard deviations, where ODn is the mean of ODs of six preimmune serum specimens.

### Cell and virus

MDCK (Madin-Darby canine kidney) cells were obtained from the American Type Culture Collection (ATCC), and maintained in Dulbecco’s minimum essential medium (DMEM; GIBCO, Invitrogen, USA) supplemented with 100 U/ml of penicillin, 100 μg/ml of streptomycin and 10% fetal bovine serum (GIBCO, Invitrogen) at 37°C in a humidified atmosphere with 5% CO_2_. Influenza A virus NIBRG-121 (NIBSC, A/California/7/2009 H1N1 virus); obtained from the CDC of Taiwan) was amplified in 10-day-old embryonic eggs at 35°C for 48 h. Virus was harvested from allantoic fluid. To determine the LD_50_ of each batch of virus, female BALB/c mice (6–7 weeks) (n = 10/group) were anesthetized subcutaneously with Zoletil 50 (Virbac Laboratories, France) (0.375 mg/mice) and inoculated intranasally with serial dilutions of the virus. The LD_50_ was the dilution of the virus that produced lethality in 50% of the mice and LD_50_ titers were calculated by the method of Reed and Muench [24]. The LD_50_ was over 10^5^ TCID50 for NIBRG-121.

### Plaque-assay

To measure virus titer, MDCK cells (5×10^6^ /well) were inoculated into 6-well microplates and were incubated at 37°C in a humidified atmosphere with 5% CO_2_ for overnight. In the second day, a serial of 10-fold dilutions of virus were prepared in PBS; MDCK cells were washed two times with PBS and 100 μl of viral dilutions were inoculated into 6-well microplates for adsorption. After one hour of adsorption, virus suspensions were removed and cells were washed two times with PBS; then 1% Oxoid agars in DMEM/BSA medium were inoculated into microwells, microplates were incubated at 37°C in a humidified atmosphere with 5% CO_2_ for three days. MDCK cells were fixed with 10% formalin for 1 h, then agars were removed and stained with crystal violet solution (0.5%) for 1 hour, stained cells were washed with tap water and were observed. Microplates were air dried at room temperature for several hours, and plaques were calculated for virus concentration.

### TCID_50_

To calculate virus titer, MDCK cells (1.5 × 10^4^ /well) in DMEM medium with 10% FBS were inoculated into 96-well microplates and were incubated at 37°C in a humidified atmosphere with 5% CO_2_ for overnight. In the second day, a serial of 10-fold dilutions of virus were prepared in PBS; MDCK cells were washed two times with PBS and 100 μl of viral dilutions were inoculated into 96-well microplates for adsorption. After one hour of adsorption, virus suspensions were removed and cells were washed two times with PBS; then DMEM/BSA medium were inoculated into microwells, and microplates were incubated at 37°C in a humidified atmosphere with 5% CO_2_ for additional five days. The TCID_50_ titers of virus were calculated by the method of Reed and Muench [24].

### Hemagglutination inhibition (HI) assay

Functional H1N1 HA-specific antibody titers were determined by HI assay using chicken erythrocytes. Prior to serological analysis, sera were treated with receptor-destroying enzyme (RDEII, Denka Seiken, Tokyo, Japan). Serum (0.1 ml) was mixed with 0.3 ml receptor-destroying enzyme, incubated at 37°C for 18 h, and adjusted to a final 1:10 dilution by adding PBS, and inactivated the enzyme activity by incubation at 56°C for 30 min. Sera giving a negative signal in the first dilution (1:10) were assigned a nominal HI score of 1:5. HI titers are expressed as reciprocal value of the highest serum dilution that inhibited hemagglutination. Animals with a serum HI titer of 1:40 were considered seroprotected.

### Intranasal influenza challenge

Four weeks after immunization with S-OIV H1N1 or NIBRG-14 H5N1 vaccine; immunized mice were blood-letting for antibody assay and were then lightly anaesthetized with Zoletil 50 (VIRBAC Laboratories, France) and challenged intranasal with 1 × 10^6^ TCID_50_ A/California/7/2009 H1N1 virus. Over the following 14 days, body weights and survival rates of each group of mice were monitored daily.

### Microneutralization assay

Neutralization antibodies of mice post vaccinated with S-OIV H1N1 or NIBRG-14 H5N1 vaccines were evaluated using microneutralization assay. Mice sera (pre-immune as negative; A/California/7/2009 NIBSC 09/152 and antiserum to NIBRG-14 H5N1 HA as positive control) were mixed with viruses (100 TCID_50_ of NIBRG-121 H1N1 or NIBRG-14 H5N1 virus) at room temperature for 1 hour, and were inoculate into 96-well MDCK cells (1.5 × 10^4^/ml). Experiment was performed following WHO protocol of microneutralization assay [21,25].

### Statistical analysis

In all figures, vertical error bars denote the standard deviation (SD). Significances of differences in antibody responses and cellular responses were evaluated by one-way analysis of variance (ANOVA). T test was used for the comparison of two specific groups in one-way ANOVA. To test the significance of survival rates between each group of immunized mice, a Z’ (alternative critical ratio) test was used [16,26]. For the comparison of HAI antibody titer, Mann–Whitney U test was used. A P value of < 0.05 was considered significant.

## Results and discussion

### Production of S-OIV H1N1 and NIBRG-14 H5N1 vaccine

It is previously reported that a single candidate seasonal H1N1 and H3N2 vaccine produced by an identical process was highly immunogenic and protective in mammalian (Vero) cells [17]. These studies were subsequently demonstrated to be highly predictive of the immunogenicity demonstrated in human trials, particularly with respect to immunogenicity at low doses and the lack of immune enhancement by use of an alum based adjuvant [27]. No clear cut correlate for protection has been established for potential pandemic vaccines such as the H5N1 vaccine or the novel H1N1 vaccine at present. Data obtained from animal protection studies could be of value in combination with data obtained from human dose finding and observational efficacy studies following vaccine use in a pandemic situation such as presently exists for the novel H1N1 virus. This study was designed to assess the immunogenicity and protective efficacy of S-OIV H1N1 and NIBRG-14 H5N1 influenza vaccines to the challenge of A/California/7/2009 H1N1 or NIBRG-14 H5N1 virus in a BALB/c mice model with a single-dose immunization regimen.

To produce a novel vaccine for S-OIV H1N1 and NIBRG-14 H5N1, NIBRG-121 and NIBRG-14 virus strains were amplified in 10-day special pathogen free (SPF) embryonic chicken eggs for 48 h and were then harvested. Viruses were inactivated with 0.04% formalin (4°C, overnight) and were purified using the Alfa Wassermann PKII Pilot-Scale Ultracentrifuge System (Alfa Wassermann Inc. (AWI), USA) Hemagglutinin (HA) antigen content was determined by single radial immunodiffusion assay. Results showed that the S-OIV H1N1 vaccine contained about 38 μg/ml of HA protein, which was estimated to be equal to 1 to 1.5 doses of vaccine (15 μg HA was regarded as one dose) for each egg. The vaccine produced was quality control tested and characterized for antigenicity (HA titer, 128–256), ovalbumin (≤ 4 μg/ml), formalin (0.373–0.548 μg/ml), and endotoxin (28–51 EU/ml) (Table 1). All tested items of vaccine produced had qualities fitting the vaccine criteria of the international standard. Furthermore, two lots of S-OIV H1N1 vaccine produced were tested for abnormal toxicity in mice. The results showed no abnormal toxicity in mice (data not shown). Results demonstrated the S-OIV H1N1 vaccines produced conformed to the quality control (QC) requirements of the World Health Organization (WHO) and European criteria for influenza vaccines. Some QC items of NIBRG-14 H5N1 vaccine did not conform to the international standard. The candidate vaccines produced were used to evaluate their protective immune effects in mice.

**Table 1:** Quality control of candidate S-OIV H1N1 and NIBRG-14 H5N1 vaccines

**Test items**	**Summary of results**	**Notes**
Abnormal toxicity	S-OIV pass, H5N1 not done	Body weight of tested mice increased
Antigenicity	HAI titer: S-OIV 128 ~ 256	Also interacted with NIBSC sntisera
	H5N1 1024	
Endotoxin	S-OIV Between 28 ~ 51,	International criteria: ≤ 200 EU/ml
	H5N1 31.4 EU/ml,	
Ovalbumin	S-OIV ≈ 4 μg/ml,	Europe: ≤ 2 μg/ml; WHO: ≤ 5 μg/ml
	H5N1 55 μg/ml	
Formaldehyde	S-OIV 0.373 ~ 0.548 μg/ml,	WHO: ≤ 0.02% ~0.01% (≈1 mg/ml)
	H5N1 not done	

Note: Result showed that all items evaluated for quality control of produced candidate vaccine fit the demand of international criteria.

Single-dose immunization of S-OIV H1N1 or NIBRG-14 H5N1 vaccine elicited a sufficient immune response to the 2009 pandemic H1N1 and avian H5N1 viruses, respectively, in a dose-dependent manner in the mice.

**Figure 1:**
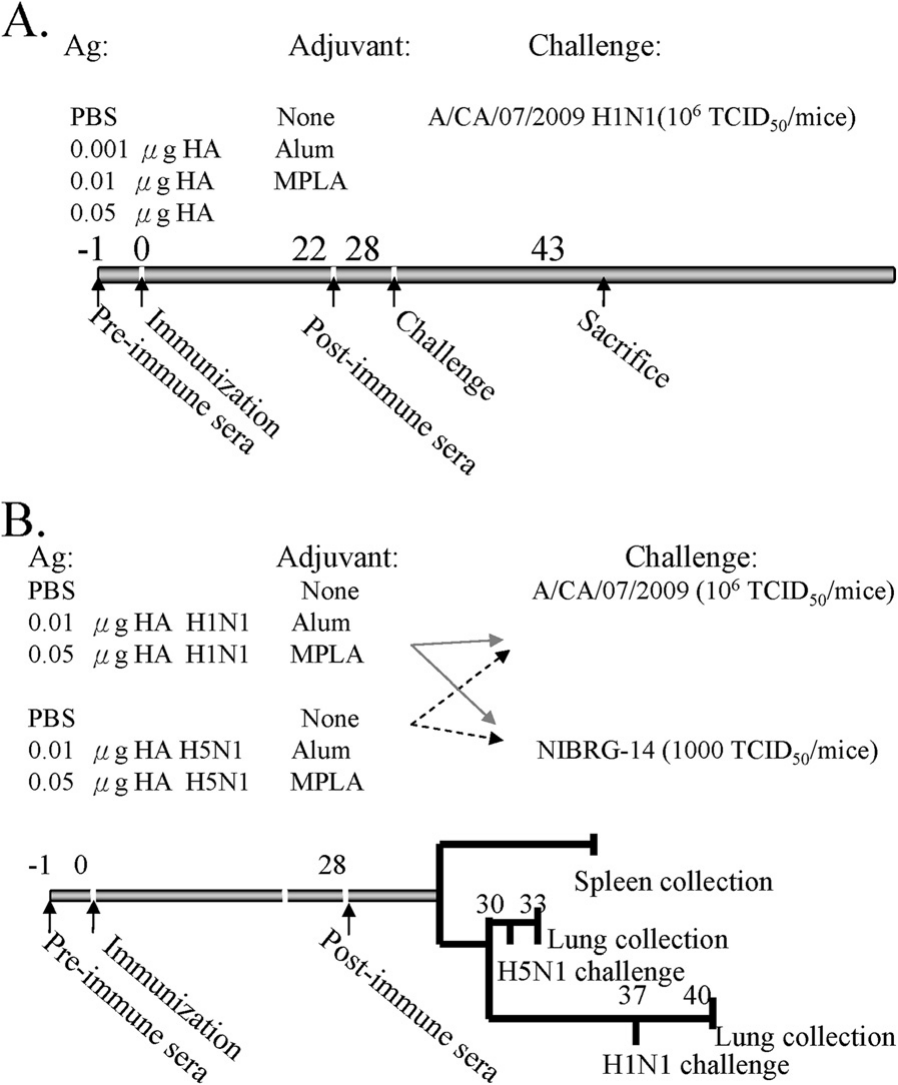
**Immunization scheme of candidate S-OIV H1N1 and avian H5N1 vaccine.** BALB/c mice were immunized with different doses of the produced vaccine S-OIV H1N1 or NIBRG-14 H5N1. (**A**) PBS control, 0.001 μg, 0.01 μg, and 0.05 μg of S-OIV H1N1 or NIBRG-14 H5N1 vaccine; (**B**) PBS control, 0.01 μg, and 0.05 μg of S-OIV H1N1 or NIBRG-14 H5N1 with or without alum or MPLA adjuvant. About 3 weeks after immunization, the mice were challenged with 10^6^ TCID_50_ of A/California/7/2009 H1N1 or 1000 TCID_50_ NIBRG-14 H5N1.

**Figure 2:**
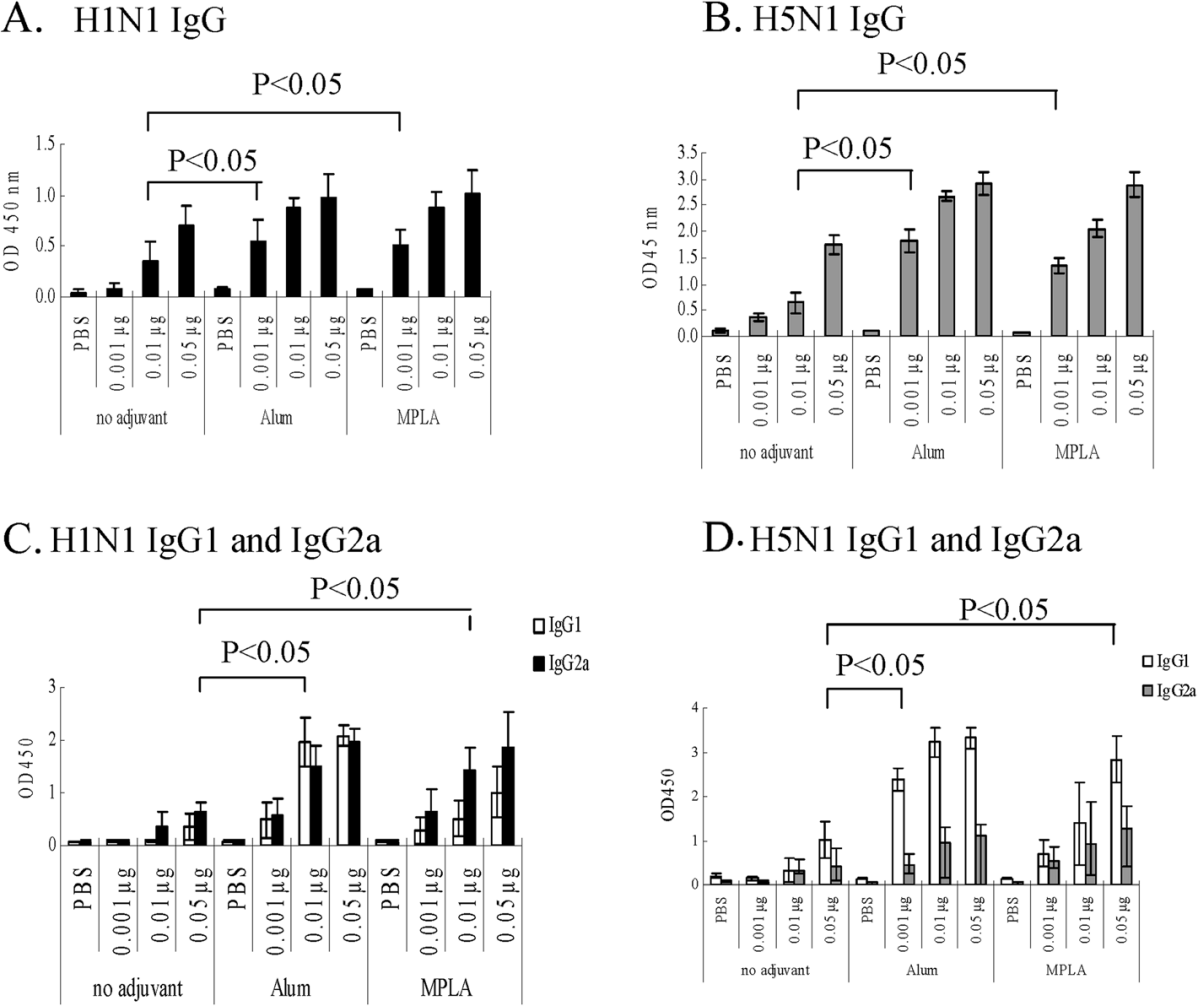
**Adjuvant promoted strong protective immune responses to S-OIV H1N1 virus.** To realize the least immunization dose of S-OIV and H5N1 vaccine needed for mice to raise sufficient protective immune responses to A/California/7/2009 virus, mice (n = 7 to 8 per group) immunized with PBS, 0.001 μg, 0.01 μg, or 0.05 μg of vaccine in the presence or absence of adjuvant. Three weeks after the single immunization, mice sera were evaluated for (**A**) H1N1 IgG, (**B**) H5N1 IgG, and (**C**) H1N1 IgG1 and IgG2a, and (**D**) H5N1 IgG1 and IgG2a immune responses to A/California/7/2009 virus. The data represent the mean titers ± SD (error bars) of antibodies in each group of animals.

In actual practical work, it seems impossible to vaccinate people twice on an emergency basis during a worldwide influenza virus pandemic. Effective vaccine components and influenza vaccine dosage for single immunization are more practical and critical for the prevention of influenza virus epidemics. In this study, a single-dose immunization of 2009 S-OIV (NIBRG-121) H1N1 and NIBRG-14 H5N1 vaccine was evaluated for its effectiveness to elicit protective and cross protective immunity in mice to the lethal challenge of A/California/07/2009 H1N1 virus (Figure 1). First, groups (10 mice per group) of mice were primed with PBS or different doses (0.001 μg, 0.01 μg, and 0.05 μg) of S-OIV H1N1 vaccine in the absence or presence of alum or MPLA adjuvant in a single-dose immunization regimen. The mice were then challenged with 10^6^ TCID_50_ of A/California/07/2009 H1N1 virus 28th days post vaccination (panel A). Second, the cross protection of S-OIV H1N1 and NIBRG-14 H5N1 vaccines were evaluated. As shown in Figure 1 panel B, the mice were immunized with PBS, 0.01 μg, or 0.05 μg of S-OIV H1N1 and NIBRG-14 H5N1 vaccines in the absence or presence of alum or MPLA adjuvant in a single-dose vaccination regimen. Mouse spleen and lungs were collected 3 days post challenge with H5N1 or H1N1 virus, respectively, and were used for microneutralization assay and mice lung viral assays. As shown in Figure 2A and 2B, S-OIV H1N1 and NIBRG-14 H5N1 vaccines elicited immune responses in a dose-dependent manner in the mice.

### Adjuvant promoted IgG responses to homologous S-OIV H1N1 and NIBRG-14 H5N1 influenza viruses

The candidate vaccine elicited an immune response in a dose-dependent manner in the mice. In the absence of adjuvant, 0.05 μg of S-OIV H1N1 and NIBRG-14 H5N1 were required to elicit a significant antibody response. When either alum or MPLA adjuvants were included in the vaccine, only 0.001 μg of S-OIV H1N1 (Figure 2A) and NIBRG-14 H5N1 vaccine (Figure 2B) were needed to significantly induce specific immune responses. The NIBRG-14 H5N1 vaccine had better antigenicity than the S-OIV H1N1 vaccine.

### Adjuvant improved S-OIV H1N1 vaccine to elicit a Th1-type antibody response (IgG2a)

**Figure 3:**
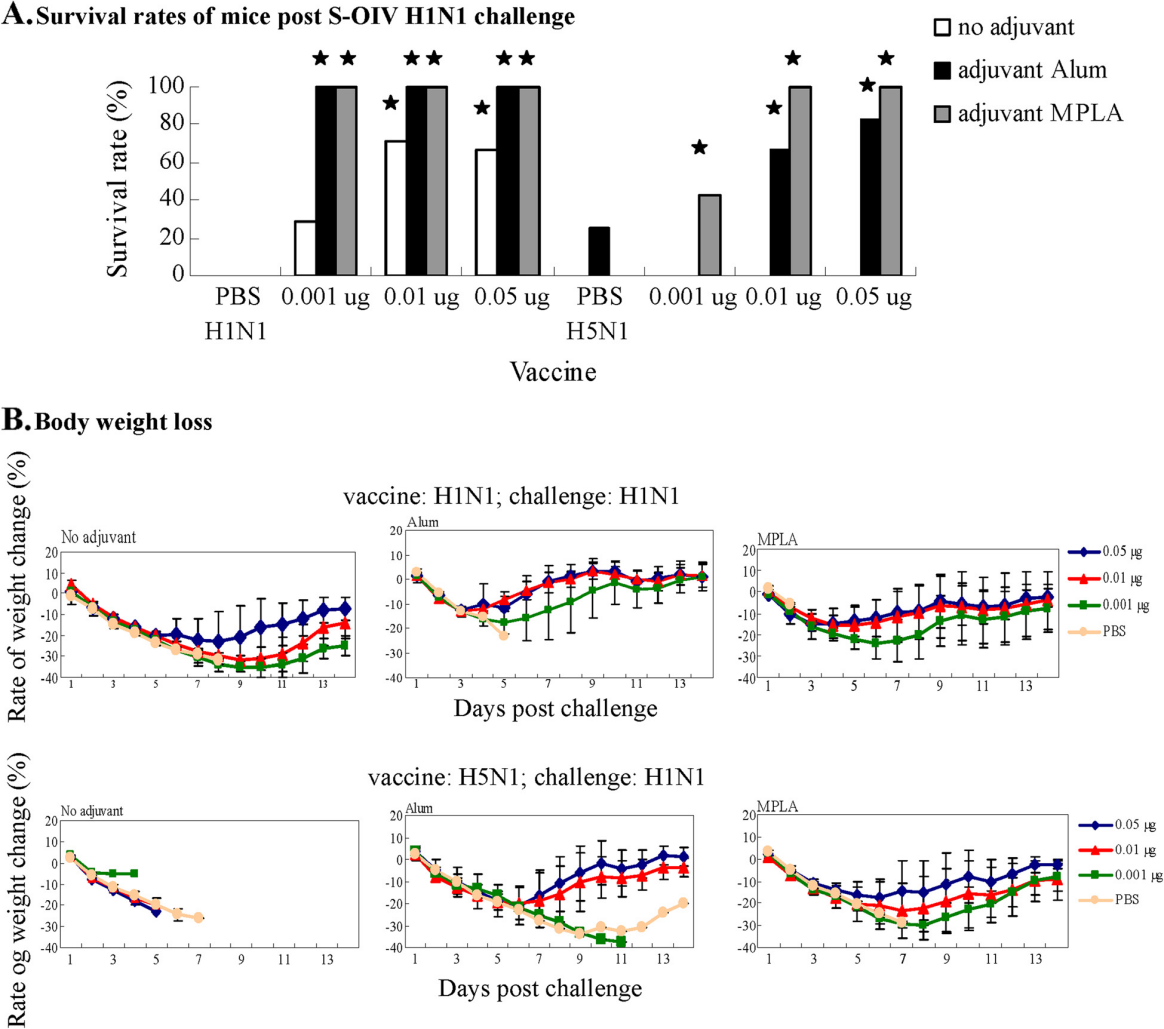
**Survival Rates and Body Weight Loss of immunized mice post challenge with A/California/7/2009 Virus.** Mice (n = 4 to 7 per group) immunized with PBS, and 0.001 μg, 0.01 μg, and 0.05 μg of vaccine with or without adjuvant (alum or MPLA), followed by lethal challenge with 10^6^ TCID_50_ of A/California/7/2009 virus intranasally. Following 14 days of observation, survival rates (**A**) and weight changes (**B**) in each group are shown.

The effects of different adjuvants were compared for their enhancement of immune response to influenza virus. Both alum and MPLA adjuvants improved the S-OIV H1N1 vaccine IgG2a immune response, an indicator of cellular immunity IgG antibody (Figure 2C). Alum adjuvant improved both humoral and cellular immune responses, while MPLA adjuvant improved the IgG2a immune response; the IgG2a/IgG1 ratio increased from 7.926 (vaccine alone) to 17.846 (vaccine plus MPLA) (Figure 2C). Controversially, both alum and MPLA adjuvants were more effective in eliciting Th2-type antibody (IgG1) responses than Th1 antibody (IgG2a) responses in mice immunized with NIBRG-14 H5N1 vaccine (Figure 2D). Thus, a candidate vaccine containing adjuvant might be able to elicit both humoral and cellular immune responses. Therefore, an S-OIV H1N1 vaccine containing alum as the adjuvant might be effective in preventing and eliminating an influenza virus infection by eliciting both Th1 and Th2 immune responses, while a vaccine containing MPLA as the adjuvant would eliminate influenza virus by enhancing mainly the Th1 immune response [6]. As shown in Figure 3A, alum and MPLA adjuvants enhanced mouse survival rates post challenge after immunization with a single-dose vaccine. These data imply that MPLA is more suitable than alum for use as an adjuvant for influenza vaccines. Another study also implied that MPLA could reduce the minimum effective immunization dosage of H5N1 and H3N2 influenza vaccines in mice [18]. For H5N1 vaccines, both alum and MPLA adjuvants effectively promoted Th2 antibody immune responses; they enhanced H5N1 vaccine-immunized mouse Th1 immune response less effectively.

### Adjuvant enhanced the vaccine’s hemagglutination inhibition (HAI) antibody response

**Figure 4:**
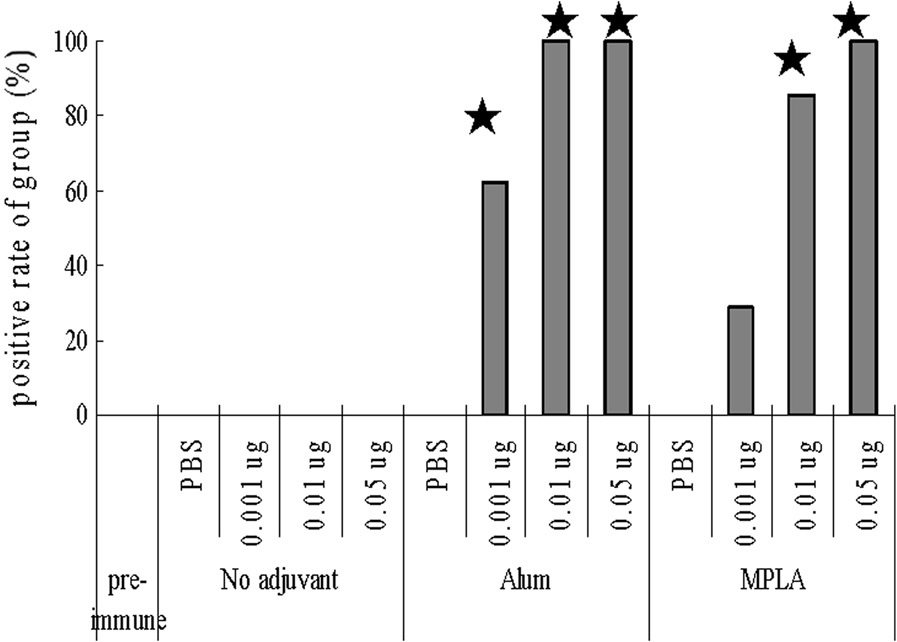
**Adjuvant enhanced vaccine to elicit a hemagglutination inhibition (HAI) antibody response.** Mice (n = 7 to 8 per group) were immunized with single different dose of S-OIV H1N1 or NIBRG-14 H5N1 influenza vaccine with or without alum or MPLA as the adjuvant. The titers of serum specific antibodies were evaluated using the hemagglutination inhibition (HAI) test. Data are representative of two separate experiments of H1N1 vaccine immunized mice sera (No sera from H5N1-immunized mice contained HAI to NIBRG-14 H5N1 virus using chicken red blood cells). Comparisons of HAI antibody titer in mice immunized with PBS, 0.001 μg, 0.1 μg, or 0.5 μg of HA in the produced vaccine with or without adjuvant. For comparison of HAI antibody titers, Student’s *t* test was used to examine the significance of differences between HAI positive rates (with HAI titer ^3^ 40) of each vaccinated group and control group (mice immunized with PBS only). A P value of < 0.05 was considered significant. The star “★” indicates significant differences.

Since the HAI antibody titer is considered an effective immunity indicator of host defense against influenza virus, we evaluated the adjuvant-induced HAI antibody response of the S-OIV H1N1 vaccine. Both alum and MPLA adjuvants enhanced HAI antibody titers when included in the 0.05-μg S-OIV H1N1 vaccine (P < 0.05) (Figure 4). Furthermore, the dosage required for the candidate S-OIV H1N1 vaccine to elicit production of a protective HAI antibody response (HAI antibody titer ≥ 40) was 0.05 μg. With this dosage, the positive rate of HAI antibody was 57.14%; the average HAI titer ranged from 80 to 160. The required vaccine dosage inducing a protective antibody response was reduced to 0.01 μg (average HAI titer of 80 to 320) or 0.001 μg (average HAI titer of 80) when the vaccines were mixed with alum or MPLA adjuvants, respectively. Similar results were also demonstrated in a study of H5N1 (NIBRG-14) inactivated whole virus and split virion influenza vaccines, in which, the use of Al(OH)_3_ with MPLA as an emulsion induced a further increase of HAI titer [12]. This implies that the addition of an adjuvant to the vaccine could reduce the vaccine dosage required to elicit a protective immune response to the S-OIV H1N1 virus.

### Minimum dosage of the S-OIV H1N1 single-dose vaccine required for generating protective immunity to a lethal challenge of a/California/07/2009 H1N1

Since a single-dose vaccination can elicit an immune response to S-OIV H1N1 virus, the minimum effective dosage of S-OIV H1N1 vaccines was evaluated. Our previous data showed that a 0.5 μg to 0.1 μg dosage of S-OIV H1N1 vaccine could provide mice with complete protection against the virus (data not shown). Therefore, groups of mice (n = 5 to 7) were immunized via the intraperitoneal (i.p.) route with different dosages of vaccine ranging from 0.05 μg to 0.001 μg in the absence or presence of alum or MPLA adjuvant. Three weeks post vaccination, the mice were bled and were challenged with A/California/07/2009 H1N1 virus one day later as described in Materials and Methods. The body weight and survival condition of the mice were recorded daily for 14 days post challenge. The vaccine provided mice protection in a dose-dependent manner (Figure 3A). Mice in the control group immunized with PBS had no protection. Single-dose vaccination of mice with the 0.05-μg candidate vaccine S-OIV H1N1 elicited sufficient protective immunity to A/California/07/2009 H1N1 challenge.

### Adjuvants reduced minimum protective dosages of both S-OIV H1N1 and NIBRG-14 H5N1 influenza vaccines

Because a large amount of influenza vaccine could be required during a pandemic or epidemic of influenza virus, an appropriate adjuvant should be considered as a component of an influenza vaccine to reduce the vaccine dosage for practical vaccination. In this study, alum and MPLA adjuvants were evaluated and were compared for their capabilities to reduce vaccine dosages required for protecting mice from a lethal challenge of S-OIV H1N1 or NIBRG-14 H5N1 influenza virus. As shown in Table 2 and Figure 3A, 0.05 μg of S-OIV H1N1 vaccine was required to yield a 65% to 85.7% survival rate upon challenge with S-OIV H1N1 virus. When alum was included as the adjuvant in the S-OIV H1N1 vaccine, the lowest dosage of vaccine for complete protection of the mice was reduced to 0.01 to 0.001 μg. Even when the mice were vaccinated with 0.001 μg of S-OIV H1N1 vaccine with alum, the survival rate was 66.67% (data not shown). Furthermore, when the adjuvant included in the vaccine was MPLA, the lowest dosage of S-OIV H1N1 vaccine providing mice complete protection from a lethal challenge of A/California/07/2009 H1N1 virus was reduced to 0.001 μg. Meanwhile, body weight loss was less significant when mice were immunized with vaccine plus alum or MPLA adjuvants than with vaccine only (Figure 3B upper panel). Body weight loss exceeded 25% of the total pre-challenge weight for mice immunized with PBS or vaccine only; while for mice immunized with vaccine plus adjuvant, body weight loss was less than 15%. Furthermore, the recovery of body weight was more rapid for mice immunized with vaccine plus adjuvant (the 5^th^ day post challenge) than for mice immunized with PBS or vaccine only (often the 7^th^ or 9^th^ day). S-OIV H1N1 vaccine accompanied by an appropriate adjuvant provided a protective effect in a single-dose immunization regimen.

**Table 2:** HAI antibody titer of mice post immunized with S-OIV H1N1 vaccine in the absence or presence of adjuvant

**Adjuvant**	**Sample dose**	**1**	**2**	**3**	**4**	**5**	**6**	**7**	**HAI ≥40**	**HAI titer**	**Mean of HAI titer**
Pre-immune		–	–	–	–	–	–	–	0/7	0	0
Non adjuvant	PBS	–	–	–	–	–			0/5	0	0
	0.05 μg	80	160	–	–	–	80	160	4/7	80 ~ 160	68.6
	0.01 μg	160	–	–	–	–	40	–	2/7	40 ~ 160	28.6
	0.001 μg	–	–	–	40	–	*	–	1/6	40	6.7
Alum	PBS	–	–	–	–	–	–	–	0/6	0	0
	0.05 μg	640	1280	80	640	1280	80	160	7/7	80 ~ 1280	594.3
	0.01 μg	160	40	80	160	160	80	320	7/7	80 ~ 320	142.9
	0.001 μg	80	–	–	–	40	80	80	4/7	40 ~ 80	40.0
MPLA	PBS	–	–	–	–	–	–	–	0/6	0	0
	0.05 μg	160	320	320	–	160	160	640	6/7	160 ~ 640	251.4
	0.01 μg	320	80	160	–	160	80	80	6/7	80 ~ 320	125.7
	0.001 μg	80	–	–	–	*	80	40	3/6	40 ~ 80	33.3

*: Did not include into calculation because of failing to immunization.

### MPLA adjuvant elicited cross-protective immunity to the lethal challenge of homogeneous and heterogeneous S-OIV H1N1 virus

Mice vaccinated with PBS or S-OIV H1N1 vaccine only did not survive the challenge with S-OIV H1N1 influenza virus (Figure 3A). Alum as an adjuvant provided some protection for mice vaccinated with 0.01 μg (60%) and 0.05 μg (80%) NIBRG-14 H5N1 vaccine upon challenged with a heterogeneous S-OIV H1N1 influenza virus. In the presence of MPLA as the adjuvant, 0.01 μg and 0.05 μg of the NIBRG-14 H5N1 vaccine provided mice complete protection to the challenge of S-OIV H1N1 influenza virus. Even vaccination with 0.001 μg of NIBRG-14 H5N1 vaccine plus MPLA as the adjuvant provided 40% survival to the challenge with S-OIV H1N1 influenza virus. Furthermnore, mice vaccinated with PBS or H5N1 vaccine alone died within 7 days post challenge with A/California/07/2009 H1N1 virus (Figure 3B lower panel). Mice vaccinated with NIBRG-14 H5N1 plus alum or MPLA as the adjuvant lost 20% to 40% or 10% to 30% of their body weight and then recovered 6 days or 7 to 8 days post challenge, respectively. MPLA not only reduced the vaccine dosage required for efficient protection from a homogeneous influenza virus, it also raised the survival rate of the lethal challenge with the heterogeneous A/California/07/2009 H1N1 virus. A previous study revealed that the addition of MPLA to the original vaccine increased CTL differentiation, and these memory cells were better equipped to rapidly kill infected cells than cells primed with alum alone [28]; another study showed that MPLA induced higher HAI antibody and IFNγ titers [18]. MPLA is a TRIF-biased agonist of TLR4 and induces expression of type I interferon through TRIF rather than MyD88 [29]. These results might be linked to the better protection of MPLA as an adjuvant; however, this remains to be elucidated.

### Adjuvant promoted IgG responses to homologous and heterogeneous influenza H1N1 and H5N1 viruses

**Figure 5:**
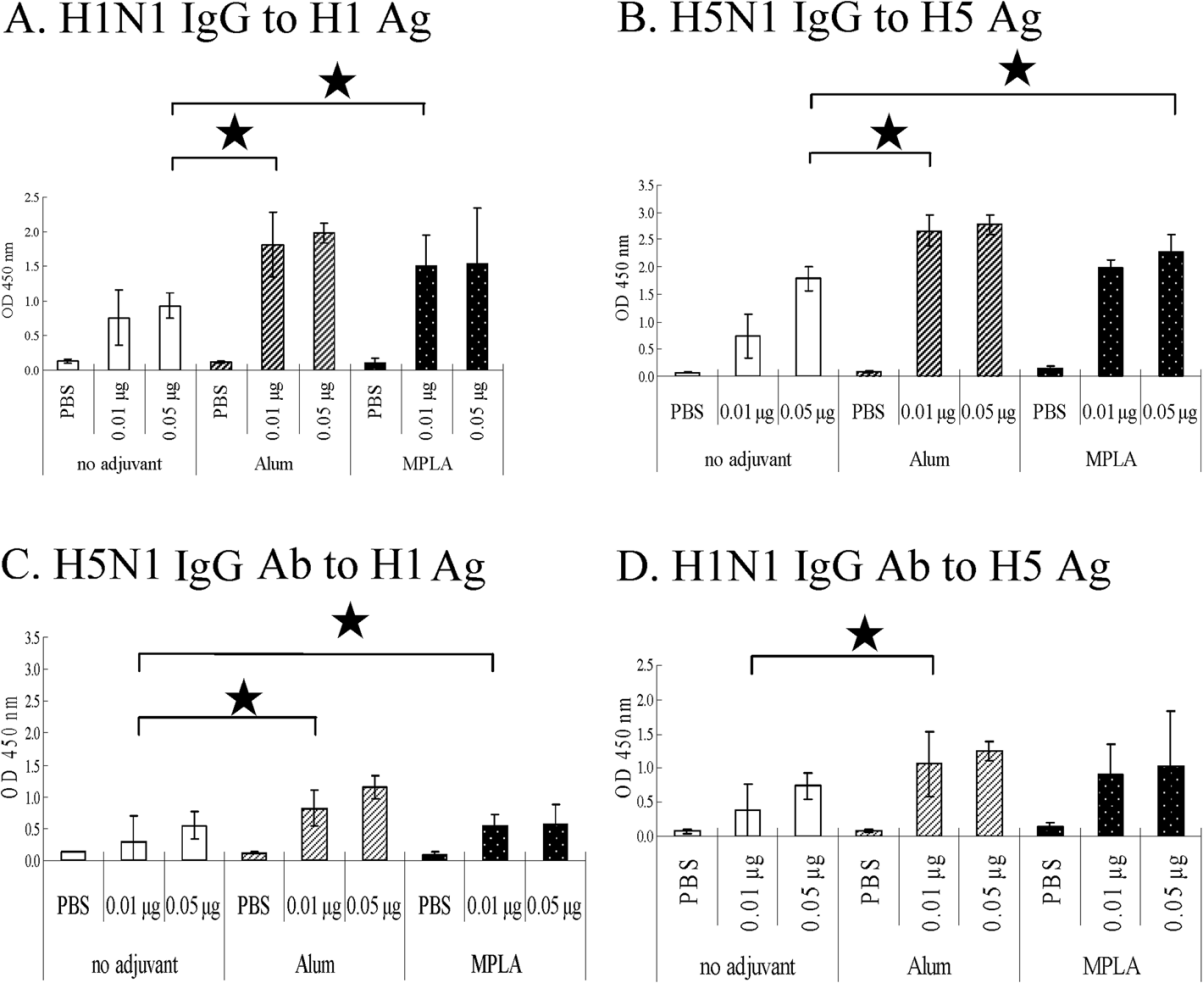
**Adjuvant promoted IgG responses of mice to homologous and heterogeneous influenza H1N1 and H5N1 virus.** To realize the least immunization dose of S-OIV and H5N1 vaccine needed for mice to raise sufficient protective immune responses to A/California/7/2009 H1N1 and NIBRG-14 H5N1 virus, mice (n = 3 to 4 per group) immunized with PBS, 0.01 μg, or 0.05 μg of vaccine with or without adjuvant. After 3 weeks of single-dose immunization, mice sera were used to evaluate the immune response type (**A**) H1N1 IgG to H1N1 virus, (**B**) H5N1 IgG to H5N1 virus, (**C**) H5N1 IgG to H1N1 virus, and (**D**) H1N1 IgG to H5N1 virus immune responses. The data represent the mean titers ± SD (error bars) of antibodies in each group of animals.

As influenza virus has antigen drift and antigen shift effects, vaccination with some influenza vaccine might not induce sufficient immunity to the threat of other influenza virus strains. In this study, we evaluated the cross protection of S-OIV H1N1 and H5N1 influenza vaccine in mice. Both alum and MPLA as adjuvants enhanced H1N1 and H5N1 homologous IgG immune responses, respectively (Figure 5A and 5B). Furthermore, the adjuvants also promoted the production of heterogeneous IgG immune responses: H5N1 IgG to S-OIV H1N1 influenza virus (Figure 5C) and H1N1 IgG to H5N1 influenza virus (Figure 5D). Alum used as the adjuvant seemed to have a better effect than MPLA did for the induction of these IgG immune responses.

### MPLA as the adjuvant significantly reduced mice lung viral titer post challenge with homologous and heterogeneous influenza viruses

**Figure 6:**
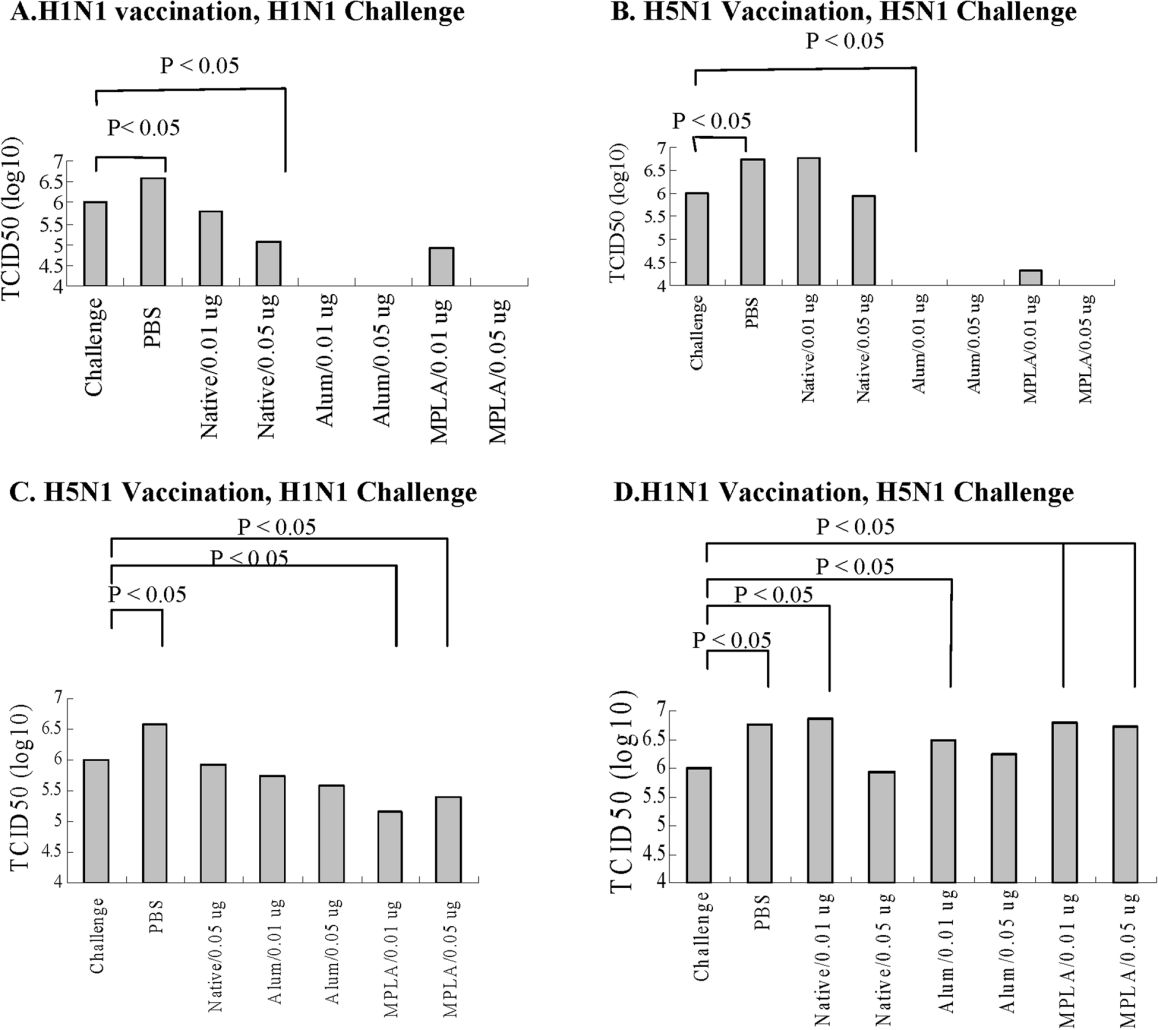
**MPLA as the adjuvant significantly reduced lung viral titer.** Mice were vaccinated with S-OIV H1N1 or NIBRG-14 H5N1 vaccines with or without different adjuvants. For the challenge, 10^6^ TCID_50_ of virus was inoculated into mice nasal cavities. Three days post challenge, mice lungs were homogenized and inoculated into MDCK cells for 48 h. Virus titers were evaluated using a micro-plaque assay. (**A**) H1N1 vaccination, H1N1 virus challenge, (**B**) H5N1 vaccination, H5N1 virus challenge, (**C**) H5N1 vaccination, H1N1 virus challenge, (**D**) H1N1 vaccination, H5N1 virus challenge. The TCID_50_ titers of the virus were calculated by the method of Reed and Muench [reference [24]. P < 0.05 indicated the significance the differences in viral titers between two groups of vaccinated mice.

To evaluate the immune responses of immunized mice to influenza virus, mice were vaccinated with S-OIV H1N1 or NIBRG-14 H5N1 vaccines in the absence or presence of different adjuvants. For the challenge, 10^6^ TCID_50_ influenza viruses were inoculated into the nasal cavities of the mice. The lungs of the mice were harvested and homogenized three days after inoculation and then, inoculated into MDCK cells and incubated for 48 h. Virus titers were evaluated using a micro-plaque assay. As shown in Figure 6A, 0.05 μg of S-OIV H1N1 vaccine significantly reduced mouse lung H1N1 viral titers; the adjuvant promoted vaccine-induced reduction of lung viral titers. For reduction of H5N1 lung viral titers, the vaccine alone had no significant effect; on the other hand, in the presence of either alum or MPLA, even 0.01 μg of NIBRG-14 H5N1 vaccine significantly reduced the lung H5N1 viral titer (Figure 6B).

To evaluate cross-protection of vaccines to S-OIV H1N1 and NIBRG-14 H5N1 influenza viruses, lung viral titers were measured in mice vaccinated with S-OIV H1N1 or NIBRG-14 H5N1 vaccines in the absence or presence of alum or MPLA included as adjuvants. NIBRG-14 H5N1 vaccine alone did not significantly reduce H1N1 viral titers in the mouse lungs (Figure 6C), where 0.01 μg of NIBRG-14 H5N1 vaccine plus MPLA significantly reduced the lung H1N1 viral titers. Conversely, S-OIV H1N1 vaccine, even with alum or MPLA adjuvant, did not significantly reduce mouse lung H5N1 viral titers post challenge (Figure 6D). Thus, the avian NIBRG-14 H5N1 might provide some cross-protection to S-OIV H1N1 virus in mice, but not vice versa. In a similar study, cross-protection of seasonal influenza virus and 2009 pandemic H1N1 influenza virus in mice and ferrets showed that vaccination with the seasonal influenza vaccines did not confer complete protection in the lower respiratory tract in either animal model, whereas the A/California/7/2009 vaccine conferred complete protection in both animal models [30]. In our study, only the MPLA adjuvant in the NIBRG-14 H5N1 vaccine reduced the heterogeneous S-OIV H1N1 lung viral titer. The underlying mechanisms remain to be elucidated.

### Adjuvant elicited homologous but not heterogeneous neutralization antibody against influenza virus

**Figure 7:**
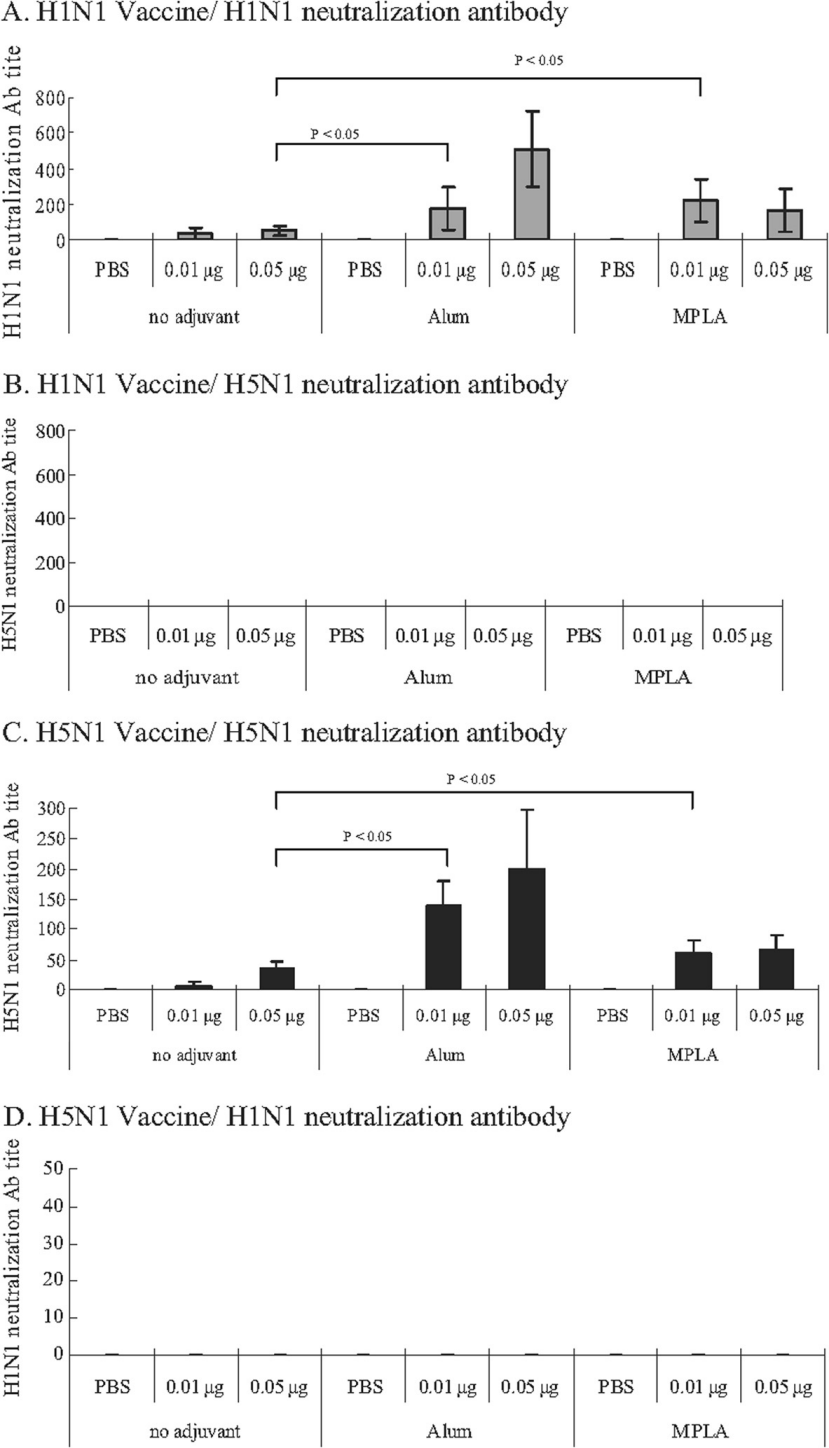
**Neutralization antibody response of immunized-mice post challenge with S-OIV H1N1 or NIBRG-14 H5N1 influenza virus.** Neutralization antibodies of mice post vaccinated with S-OIV H1N1 or NIBRG-14 H5N1 vaccines were evaluated using a microneutralization assay. Mice sera (pre-immune as negative; A/California/7/2009 NIBSC 09/152 and antiserum to NIBRG-14 H5N1 HA as positive control) were mixed with viruses (100 TCID_50_ of NIBRG-121 H1N1 or NIBRG-14 H5N1 virus) at room temperature for 1 hour, and were inoculated into 96-well MDCK cells (1.5 × 10^4^/ml). Experiments were performed following the WHO protocol for the microneutralization assay. (**A**) H1N1 vaccination, H1N1 neutralization antibody, (**B**) H5N1 vaccination, H5N1 neutralization antibody, (**C**) H5N1 vaccination, H1N1 neutralization antibody, (**D**) H1N1 vaccination, H5N1 neutralization antibody. Error bar is the standard deviation of six serum samples.

Neutralization antibody always plays an important role in the evaluation of host immunity after vaccination for influenza viruses. We evaluated the roles of adjuvants in the vaccines to elicit neutralization antibody. H1N1 and H5N1 vaccines with alum or MPLA adjuvant elicited significantly more homologous neutralization antibodies to S-OIV H1N1 and H5N1 influenza virus, respectively, than vaccine alone (Figure 7A and 7C). Alum enhanced the production of neutralization antibody against homologous influenza virus, but none of adjuvants induced production of heterogeneous neutralizing antibody (Figure 7B and 7D).

Our results indicate that a significant reduction of influenza viral titer in lungs of mice three days post challenge was a better indicator for survival prediction than hemagglutination inhibition (HAI) and neutralization antibody titers. Alum and MPLA used as adjuvants reduce the vaccine dosage requirement and elicit protective immunity to homologous influenza virus. MPLA further promoted vaccine cross-protection in mice vaccinated with NIBRG-14 H5N1 to lethal challenge with heterogeneous S-OIV H1N1 influenza virus.

## Conclusion

The study results showed that a single-dose immunization in BALB/c mice with 0.1 μg of the candidate vaccine elicited complete protective immunity to S-OIV H1N1 virus. This result is consistent with a previous study [16]; this minimal effective vaccine dose in BALB/c mice corresponds to approximately 30 μg of HA in humans. Furthermore, we also learned that when the vaccine contained an appropriate amount of MPLA adjuvant, the vaccine elicited protective immunity to S-OIV H1N1 with single-dose immunization using 1/100 to 1/10 of the original vaccine dosage (corresponding to approximately 0.3 to 3 μg of HA in humans). Additionally, both H1N1 and H5N1 induced significant homologous, but not heterogeneous neutralization antibody. Only in the presence of adjuvant could the influenza vaccine protect mice from a lethal challenge of heterogeneous influenza virus. MPLA as the adjuvant with H5N1 vaccine significantly reduced lung H1N1 virus titers post challenge in the mice. Results revealed that lung viral titer was a better indicator than IgG, HAI, and neutralization antibodies titer, in predicting survival rates of mice post influenza virus challenge. Results from this study revealed (1) the minimum protective vaccination dosage; (2) the single-dose vaccination regimen induced protective immunity in the presence of adjuvant; (3) cross-protection evaluation between S-OIV H1N1 and NIBRG-14 H5N1 vaccines; (4) adjuvant promoted antiviral protection and reduce the required vaccination dosage; and (5) MPLA as the adjuvant promoted better cross-protection of NIBRG-14 H5N1 vaccine to the lethal challenge with the 2009 pandemic H1N1 influenza virus, possibly through Th1 immunity. These data provide important insight for the design and development of vaccine formulas and adjuvants.

## Competing interests

The authors declare that they have no competing interests.

## Authors’ contributions

LHT carried out overall research work; CCC carried vaccine preparation and animal experimental; WHL carried out animal experimental; CDM supervised the overall progress; WYC is responsible for the overall research. All authors have read and approved the final manuscript.
